# Brain omega-3 polyunsaturated fatty acids modulate microglia cell number and morphology in response to intracerebroventricular amyloid-β 1-40 in mice

**DOI:** 10.1186/s12974-016-0721-5

**Published:** 2016-09-29

**Authors:** Kathryn E. Hopperton, Marc-Olivier Trépanier, Vanessa Giuliano, Richard P. Bazinet

**Affiliations:** Department of Nutritional Sciences, Faculty of Medicine, University of Toronto, FitzGerald Building, 150 College St., Room 306, Toronto, ON M5S 3E2 Canada

**Keywords:** Alzheimer’s disease, Omega-3 polyunsaturated fatty acids, Neuroinflammation, Microglia, Amyloid-β

## Abstract

**Background:**

Neuroinflammation is a proposed mechanism by which Alzheimer’s disease (AD) pathology potentiates neuronal death and cognitive decline. Consumption of omega-3 polyunsaturated fatty acids (PUFA) is associated with a decreased risk of AD in human observational studies and exerts protective effects on cognition and pathology in animal models. These fatty acids and molecules derived from them are known to have anti-inflammatory and pro-resolving properties, presenting a potential mechanism for these protective effects.

**Methods:**

Here, we explore this mechanism using fat-1 transgenic mice and their wild type littermates weaned onto either a fish oil diet (high in n-3 PUFA) or a safflower oil diet (negligible n-3 PUFA). The fat-1 mouse carries a transgene that enables it to convert omega-6 to omega-3 PUFA. At 12 weeks of age, mice underwent intracerebroventricular (icv) infusion of amyloid-β 1-40. Brains were collected between 1 and 28 days post-icv, and hippocampal microglia, astrocytes, and degenerating neurons were quantified by immunohistochemistry with epifluorescence microscopy, while microglia morphology was assessed with confocal microscopy and skeleton analysis.

**Results:**

Fat-1 mice fed with the safflower oil diet and wild type mice fed with the fish oil diet had higher brain DHA in comparison with the wild type mice fed with the safflower oil diet. Relative to the wild type mice fed with the safflower oil diet, fat-1 mice exhibited a lower peak in the number of labelled microglia, wild type mice fed with fish oil had fewer degenerating neurons, and both exhibited alterations in microglia morphology at 10 days post-surgery. There were no differences in astrocyte number at any time point and no differences in the time course of microglia or astrocyte activation following infusion of amyloid-β 1-40.

**Conclusions:**

Increasing brain DHA, through either dietary or transgenic means, decreases some elements of the inflammatory response to amyloid-β in a mouse model of AD. This supports the hypothesis that omega-3 PUFA may be protective against AD by modulating the immune response to amyloid-β.

## Background

Alzheimer’s disease (AD) is characterized by neuronal loss, the deposition of amyloid-β plaques, and the hyperphosphorylation of intracellular tau proteins, leading to the formation of neurofibrillary tangles. In addition to these features, neuroinflammation is increasingly recognized as a hallmark of AD. Postmortem studies have detected higher levels of neuroinflammatory markers, such as astrocytes, microglia, cytokines, or complement, in the brains of patients with AD than in controls [1–6]. Patients with AD have higher plasma levels of cytokines, such as interleukin (IL)-6 and IL-1β [7], than healthy controls, while in patients with mild to moderate AD, elevation in serum tumor necrosis factor (TNF)-α is associated with cognitive decline [8]. Positron emission tomography studies using ligands to the peripheral benzodiazepine binding receptor (such as [^11^C](R)-PK11195), which is thought to label activated microglia, show higher binding in AD patients than in controls [9] which co-localizes with binding of [^11^C] Pittsburgh compound B (PIB), a marker of fibrillary amyloid-β [10]. Interestingly, scores on the mini mental state exam, a measure of cognitive impairment where lower scores indicate greater impairment, are inversely correlated with PK11195 binding, but not with uptake of PIB [10], suggesting an independent effect of inflammation on cognitive decline. This is supported by studies associating genetic polymorphisms in various inflammation-associated genes with AD risk, including polymorphisms in triggering receptor expressed on myeloid cell (TREM) 2, cluster of differentiation (CD) 33, IL-6, toll-like receptor (TLR) 4, and IL-1 [11–17].

Elevations in markers of neuroinflammation have also been identified in animal models of AD, such as higher levels of IL-1β and chemokine CXCL motif (CXCL) ligand 1 in TgCRND8 mice than their wild type littermates [18] and increases in TNF-α, monocyte chemoattractant protein (MCP)-1, and microglia in 3xTg mice compared to controls [19]. In intracerebroventricular (icv) infusion AD models, in which amyloid-β is injected into the brains of rodents either acutely or chronically via a pump, there are elevations in brain cytokines, TNF-α and IL-1β [20], and glial fibrillary acidic protein (GFAP) and CD68, markers of astrocytes and microglia, [21] relative to controls. Treatments that decrease neuroinflammatory markers in animal models generally improve behavioral scores and decrease AD pathology [22–25]. Interestingly, immune activation has been shown to increase the production of amyloid-β and the hyperphosphorylation of tau proteins [26] and seems to precede the deposition of amyloid-β plaques [27], which supports the hypothesis that inflammation is a causal factor in AD development (for review, see [28]).

Docosahexaenoic acid (DHA), the main omega-3 polyunsaturated fatty acid (PUFA) in the brain, may be protective in AD through several mechanisms (for review, see [29]). DHA promotes neuronal development and synaptogenesis through its conversion to synaptamide (docosahexaenoyl ethanolamide), a member of the endocannabinoid family [30]. DHA also regulates levels of brain-derived neurotrophic factor [31], which could protect against neuronal and synaptic loss. DHA and eicosapentaenoic acid (EPA) exert immuno-modulatory effects and are precursors to a class of bioactive lipid molecules, referred to as specialized pro-resolving mediators, that have well-characterized anti-inflammatory and pro-resolving properties (for review, see [32]). These mediators may also exert neuroprotective effects, as neuroprotection D1, a specialized pro-resolving mediator derived from DHA, upregulates the expression of anti-apoptotic genes such as Bcl-2 [33]. Two studies in postmortem human brain samples detected lower levels of specialized pro-resolving mediators, including maresin 1, protectin D1, and resolvin D5 in the hippocampus [33] and entorhinal cortex [34] of patients with AD relative to controls, suggesting that impairments in resolution of inflammation may be involved in this disease.

Fish consumption and/or elevated blood levels of omega-3 PUFA are associated in human observational studies with a decreased risk of AD and a slower rate of cognitive decline [35, 36]. A recent meta-analysis of animal studies identified improvements in amyloid-β plaque levels, cognition, and neurodegeneration in AD models with omega-3 PUFA treatment [37]. In contrast, human intervention studies in AD are generally null [38]; however, there is some evidence of protection in more mild forms of the disease [39]. As pathological features of AD may develop over decades prior to the appearance of symptoms [40], the discrepancy between the results of the epidemiologic studies, which are mostly primary prevention, and clinical trials in patients with diagnosed AD may be explained by: differences in the magnitude of pathology in these populations, by the possibility of a critical window for effectiveness of a dietary intervention or by residual confounding. Six animal studies have measured an inflammatory outcome in an AD model following interventions aimed at increasing brain omega-3 PUFA. These are summarized in Table 1, (updated from [41]). Two studies fed rats with EPA for 4 weeks and noted reductions in hippocampal protein levels of interferon (IFN)-γ and IL-1β and increases in peroxisome proliferator-activated receptor (PPAR)γ compared to control-fed animals 3 h following icv infusion of amyloid-β 1-40 [42, 43]. Another two studies used the same icv model but fed EPA [44] or DHA [45] for 27 days and identified dose-dependent reductions in the hippocampal mRNA and protein for CD11b, GFAP, IL-1β, and TNF-α 7 days following icv infusion of amyloid-β relative to rats consuming the control chow. One study crossed triple transgenic 3xTg-AD mice with fat-1 mice, a transgenic animal expressing an omega-3 desaturase gene that allows it to convert omega-6 to omega-3 fatty acids, and detected lower levels of GFAP protein in the cortex of 3xTg-AD mice expressing the fat-1 gene after 18 months [46]. In contrast, Parrott et al. noted a deterioration in cognitive functioning and an increase in hippocampal gene expression of TNF-α when TgCRND8 mice were fed a whole food diet containing freeze-dried powdered fish, fruits, and vegetables [47]. As the diet contained multiple interventions, it cannot be determined whether this increase in inflammatory markers is attributable to the fish feeding [47].

**Table 1:** Studies examining neuroinflammatory markers in AD models following omega-3 interventions

Author (year)	AD model	Species	Omega-3 PUFA treatment	Timing of inflammation measurement	Inflammatory outcome
Minogue (2007) [43]	icv aβ 1-40	Rat	125 mg/day EPA vs MUFA × 4 weeks	3 h post-surgery	↓ IFN-γ, IL-1β protein
Lynch (2007) [42]	icv aβ 1-40	Rat	125 mg/day EPA vs MUFA × 4 weeks	3 h post-surgery	↓ IL-1β protein
Lebbadi (2014) [46]	3xTg-AD	Mouse	Fat-1 cross	12 or 20 months old	↓ GFAP,↔iPLA_2_, cPLA_2_ protein
Parrott (2015) [47]	TgCRND8	Mouse	Whole food diet containing salmon, fruits and vegetables 2.46 mg DHA/g diet	After 7 months feeding	↑ TNF-α mRNA
Wen (2016) [44]	icv aβ 1-40	Rat	150 or 300 mg/kg/day EPA × 27 days	13 days post-surgery	↓ CD11b, GFAP, TNF-α, IL-1β mRNA and protein
Wen (2016) [45]	icv aβ 1-40	Rat	300 mg/kg/day DHA-PS or DHA-PC × 27 days	27 days post-surgery	↓ CD11b, GFAP, TNF-α, IL-1β mRNA and protein

*Aβ* amyloid-β*, CD* cluster of differentiation, *DHA* docosahexaenoic acid, *EPA* eicosapentaenoic acid, *GFAP* glial fibrillary acidic protein, *icv* intracerebroventricular, *IFN* interferon, *IL* interleukin, *MUFA* monounsaturated fatty acid, *PC* phosphatidylcholine, *PLA* phospholipase A, *PS* phosphatidylserine, *TNF* tumor necrosis factor

As markers of inflammation are produced dynamically in response to an insult, with an initial increase in levels followed by resolution (a return to homeostasis), examining neuroinflammatory markers over time can be useful to understand how inflammation and its resolution are affected by omega-3 PUFA. Omega-3 PUFA may affect neuroinflammation by decreasing the peak in production of some markers but not others, or by shifting the time course of their production in ways that are not captured by measurements at a single time point [41]. As inflammation in the brain is mainly controlled by a different set of cells than occur in the periphery, the astrocytes and microglia, we set out first to characterize the time course of the inflammatory response to amyloid-β 1-40, and then to see how this was affected by changing brain levels of DHA through a dietary or transgenic approach. While we identified no effect of changing brain DHA on astrocytes, we detected a lower peak in microglia activation in the hippocampus of mice with elevated brain DHA, along with reductions in markers of neurodegeneration and alterations in microglia morphology that may be indicative of a less activated phenotype.

## Methods

### Animals

All animal procedures and husbandry were carried out in accordance with the Regulations of Animals for Research Act in Ontario and the Guidelines of the Canadian Council on Animal Care (2015/16 protocol numbers 20011375 and 20011376). Mice were housed in the University of Toronto Department of Comparative Medicine animal facility at a controlled temperature (21 °C) and light cycle (14/10 light/dark), 1–4 per cage with ad libitum access to food and water.

In the first study, 10-week-old male C57BL/6 mice were obtained from Charles River Laboratories (Saint Constant, Quebec, Canada) and were maintained on standard laboratory chow both during a 2 week acclimatization period prior to surgery and following surgery until death.

Mice for the second study were obtained via breeding in-house from male fat-1 mice provided as a generous gift by Dr. David Ma (University of Guelph, ON, Canada). The fat-1 mouse carries a *fat-1* transgene from the roundworm *Caenorhabditis elegans*, enabling it to endogenously convert omega-6 to omega-3 PUFA, and thus attain high tissue levels of omega-3 PUFA on an omega-3 PUFA deplete diet [48]. C57BL/6 dams were ordered from Charles River Laboratories at 5–6 weeks of age and maintained on the low omega-3, 10 % safflower oil (SO) diet for 2 weeks prior to breeding with fat-1 males. Dams were maintained on the SO diet throughout the pregnancy and lactation to reduce maternal transfer of omega-3 PUFA. Fat-1 mice were weaned onto the SO diet, while the wild type (WT) offspring were weaned onto either the SO diet or a diet that contained 8 % safflower oil and 2 % fish oil (FO). Offspring were maintained on these diets until 12 weeks of age, at which point they underwent icv surgery and were returned to the same diets after surgery until death.

### Diets

Animals were fed one of the three experimental diets depending on the study: standard laboratory chow (Teklad 2018, Envigo, Indianapolis, IN, USA) or one of two diets modified from the AIN-93G rodent diet: the SO diet, which contains 10 % safflower oil by weight (SO; D04092701; Research Diets Inc., New Brunswick, NJ, USA), or the FO diet which contains 2 % menhaden oil and 8 % safflower oil (FO; D04092702; Research Diets Inc.). The fatty acid composition of the chow diet used in our facility has been reported previously [49] and contains 53 % linoleic acid, 19 % oleic acid, 15 % palmitic acid, 6 % alpha linolenic acid (ALA), and trace amounts (<0.5 %) of other fatty acids. Fatty acid composition of the FO and SO diets was confirmed in triplicate on both fresh (sampled from a sealed box stored at 4 °C) or week-old (sampled from hoppers in the animal facility after at least 1 week at room temperature) pellets. The main fatty acid species in fresh samples of the two diets are shown in Table 2. As a percent of fatty acids, the most abundant fatty acids in the SO diet are linoleic acid (18:2n-6, 70.7 %), oleic acid (18:1n-9, 15.5 %), palmitic acid (16:0, 8.0 %), and stearic acid (18:0, 2.9 %). The main fatty acid species of the FO diet are linoleic acid (59.9 %), oleic acid (13.6 %), palmitic acid (10.3 %), myristic acid (14:0, 2.7 %), EPA (20:5n-3, 2.6 %), and DHA (22:6n-3, 1.5 %). Neither diet contained >0.5 % of any other fatty acid not listed in Table 2, and neither diet’s measured composition differed from the manufacturer’s product specifications or what has been measured in our lab previously [50]. The fatty acid composition of the fresh and week-old diets also did not differ.

**Table 2:** Fatty acid composition of 10 % safflower oil and 2 % fish oil, 8 % safflower oil diets

	10 % Safflower oil	2 % Fish oil, 8 % safflower oil
Fatty acid composition		
14:0	n.d	2.7
16:0	8.0	10.3
16:1n-7	n.d	3.1
18:0	2.9	2.8
18:1n-9	15.5	13.6
18:1n-7	0.7	1.1
18:2n-6	70.7	59.9
18:3n-3	0.5	0.8
EPA	n.d	2.6
DHA	n.d	1.5

Fatty acid percent compositions are calculated as the percentage of the total identified fatty acids and are means of triplicate analysis. Other fatty acids are present at levels <0.5 % of total fatty acids not shown

*n.d.* not detected

### Genotyping

Genotyping was carried out using a method adapted from Orr et al*.* [50]. Tails of 2–3-week-old mice were coated with EMLA analgesic cream (AstraZeneca, Mississauga, Canada), after which 2–3 mm of the tip of the tail was removed and the wound cauterized. Tails were digested overnight in a cell lysis buffer (100 mM Tris HCl pH 8.5, 5 mM EDTA, 0.2 % sodium dodecyl sulfate, 200 mM NaCl) with 0.8 mg/ml proteinase K. Tail debris was pelleted (20 min × 15,700 relative centrifugal force (rcf)), and DNA was precipitated by eluting the supernatant into 1 ml isopropanol. DNA was pelleted (10 min × 15,700 rcf), and the supernatant was removed to allow the pellet to dry. The pellet was then resuspended in ×1 Tris-EDTA buffer. One −1.5 μl of DNA was used in a PCR reaction with a commercial mastermix (Thermo Scientific, Waltham, MA, USA) as per manufacturer’s instructions with the following PCR conditions: 2 min × 95 °C, 30 cycles × (30 s 94 °C, 30 s 55 °C, 1 min 72 °C), followed by the final elongation step for 10 min at 72 °C. Resultant 250 base pair bands were visualized on a 1.5 % agarose gel containing SYBR Safe DNA Gel Stain (Life Technologies, ThermoScientific, Waltham, MA, USA) using a UV light box.

### Gas chromatography

A separate group of non-surgery mice were killed by CO_2_ asphyxiation at 12 weeks of age, and total lipids were extracted from whole brains using a method adapted from Folch et al*.* [51]*.* Total fatty acids were measured and quantified as described in detail by our lab previously [52].

### Preparation of amyloid-β 1-40 and 40-1 injections

**Fig. 1:**
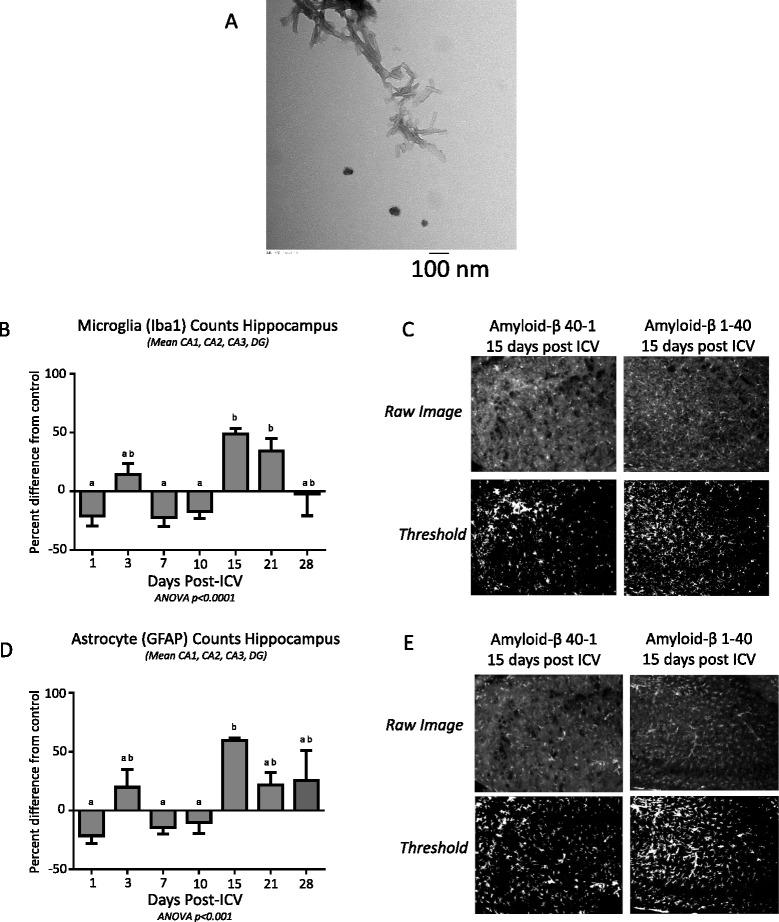
**a** TEM image of aggregated amyloid-β 1-40, length of 100–500 nm and smooth appearance characteristic of fibers. **b** Mean ± SEM of iba1-labelled microglia counts in the CA1, CA2, CA3, and DG of the hippocampus following intracerebroventricular infusion of amyloid-β 1-40, normalized for the counts following infusion of control peptide (amyloid-β 40-1). **c** Sample images of the CA3 region of the hippocampus 15 days following intracerebroventricular infusion of either amyloid-β 1-40 (*right*) or the control peptide amyloid-β 40-1 (*left*). **d** Mean ± SEM of GFAP-labelled astrocyte counts in the CA1, CA2, CA3, and DG of the hippocampus following intracerebroventricular infusion of amyloid-β 1-40, normalized for the counts following infusion of control peptide. **e** Sample images from the CA3 region of the hippocampus 15 days following intracerebroventricular infusion of either amyloid-β 1-40 (*right*) or the control peptide amyloid-β 40-1 (*left*). The *top row* of images in **c** and **e** is enhanced for publication, while the *bottom row* of images is the same image in which a threshold was applied to show labelled cells. *Different letters* denote significant differences (*p* < 0.05) by one-way ANOVA followed by Bonferroni post hoc test. *CA* cornu ammonis, *DG* dentate gyrus, *GFAP* glial fibrillary acidic protein, *iba1* ionized calcium-binding adapter molecule 1. *SEM* standard error of the mean *TEM* transmission electron microscopy

Amyloid-β 1-40 and a reverse peptide control, amyloid-β 40-1, were obtained from Bachem Biochemicals (H-1194 and H-2972, respectively; Bachem Biochemicals, Bubendorf, Switzerland). The lyophilized powder was diluted to 1 μg/μl in sterile 0.1 M phosphate-buffered saline (PBS) and aggregated at 37 °C for 96 h to promote formation of oligomers, fibrils, and fibers as described previously [20, 21, 53]. Aggregation was confirmed by electron microscopy (Fig. 1a) by identifying fibrils 100–500 nm long and smooth in appearance [54, 55]. Treatment and control solutions were aliquoted and stored at −20 °C until use.

### Negative-stain transmission electron microscopy

Electron microscopy was conducted according to published methods [56]. Briefly, 1 % Pioloform-coated copper grids (Canemco #G300HEX, Canada) were charged using a glow discharge apparatus (Quorum Technologies, Laughton, East Sussex, UK) at 0.15 Torr × 15 s, 5 mA, and 2 μl of 1 μg/μl amyloid-β 1-40 in PBS was loaded for 1 min, wicked to remove large solvent droplets and allowed to air dry. Two microliter of 1 % phosphotungstic acid were then loaded for 45 s to stain the grids, then wicked and allowed to air dry under an incandescent light bulb. Grids were then loaded into a grid deck and visualized via a transmission electron microscope (Hitachi H-7000 TEM, Japan) at 75 kV.

### Intracerebroventricular amyloid-β infusion surgery

Surgeries were conducted as described previously [49]. Mice were anesthetized by isoflurane (induction 3 %, maintenance 2 %) and the top of head shaved. The head was secured via ear and teeth bars in a stereotaxic setup with a digital reader (Stoelting, Wood Dale, IL, USA). The analgesic Marcaine (Hospira Healthcare Corporation, Montreal, Québec, Canada) was injected at 1.5 mg/kg subcutaneously at the incision site. After 5 min, the skull was exposed and the digital reader was calibrated to the bregma. The head was gently raised or lowered to ensure the skull was level (<0.1 mm difference in height between bregma and lambda). A small hole was then drilled −1.0 mm medial/lateral and −0.5 mm anterior/posterior to bregma, and a 33-gauge needle was lowered −2.4 mm dorsal/ventral into the left lateral ventricle. Five microliter of amyloid-β 1-40 or 40-1 was then infused at a rate of 1 μl/min via a quintessential stereotaxic injector (Stoelting). The needle was kept in the ventricle for 25 min post-infusion to ensure treatment diffusion in the cerebrospinal fluid before being slowly raised to prevent backflow. The accuracy of this injection into the lateral ventricle was checked periodically by injection with Evan’s blue dye. The hole in the skull was sealed with bone wax (Ethicon, Somerville, New Jersey, USA) and the scalp sutured shut. Mice were monitored post-surgery until autonomous head movement was recovered and were housed singly until death. Mice were euthanized at various time points between 24 h and 28 days post-surgery. Time points were selected based on previous work in our lab that found that microglia and astrocyte activation following icv lipopolysaccharide (LPS) began increasing after 24 h (unpublished) and on preliminary experiments described here.

### Sample preparation and immunohistochemistry

Mice were anesthetized with 250 mg/kg intraperitoneal avertin and euthanized via transcardiac perfusion with cold PBS for 3 min, followed by 7 min of 4 % paraformaldehyde, infused at a rate of 4 ml/min using a peristaltic pump (GE Healthcare, Mississauga, ON, Canada). Brains were extracted and post-fixed for 24 h in 4 % paraformaldehyde, followed by dehydration and storage in a 30 % sucrose solution until sectioning. Brains were frozen in Cryomatrix sectioning medium (Thermo Scientific, Waltham, MA, USA) and sliced into 40 μm sections using a cryostat (Leica, CM 1510S, Concord, ON). Slices were stored in 0.05 % sodium azide until analysis.

For immunohistochemistry to visualize astrocytes and microglia, slices were washed three times for 10 min each in PBS and quenched for 10 min in 0.5 % sodium borohydride, followed by another three PBS washes. Sections were blocked for 2 h in a solution of 10 % normal goat serum, 0.75 % bovine serum albumin, and 0.1 % Triton-X in PBS and incubated overnight in antibody solution (2 % normal goat serum, 0.01 % Triton-X in PBS), with rabbit anti-ionized calcium-binding adapter molecule 1 (iba1) (Wako Chemicals, Richmond VA, USA) and mouse anti-GFAP (Antibodies Inc., Davis, CA, USA) antibodies. Anti-iba1 was diluted to a concentration of 1:2000 for epifluorescent microscopy and 1:1000 for confocal microscopy, while GFAP was diluted 1:1000 for epifluorescent microscopy and 1:500 for confocal microscopy. Slices were washed three times in cold PBS, and then incubated for 1 h in antibody solution with 1:2000 goat anti-rabbit Alexa Fluor 568 and 1:2000 goat anti-mouse Alexa Fluor 488 (Life Technologies, Burlington, ON, Canada). Slices were then washed three times in PBS and mounted onto glass microscope slides in VECTASHIELD Antifade Mounting Medium with DAPI (Vector Laboratories, Bulingame, CA, USA) and coverslipped with #1 type micro cover glasses (VWR International, Mississauga, ON, Canada).

Fluoro Jade C (FJC; Millipore, Darmstadt, Germany) immunohistochemistry was used to visualize degenerating neurons via a method adapted from the manufacturer’s specifications. Whole brain coronal sections were washed three times in PBS, mounted onto poly l-lysine-coated slides (Sigma-Aldrich, Oakville, ON Canada) and allowed to dry overnight. Slides were then placed in a staining rack and moved sequentially through the following solutions: dH_2_O × 1 min, 100 % ethanol × 3 min, 70 % ethanol × 1 min, dH_2_O × 1 min, 0.06 % potassium permanganate × 15 min on a shaker, dH_2_O × 1 min, and then avoiding light: 0.001 % FJC + 0.2 % Hoescht stain in 0.1 % acetic acid × 30 min followed by 3 × 1 min washes in dH_2_O prior to drying overnight in the dark.

### Epi-fluorescence microscopy and cell counting

Cells were visualized in the hippocampus, stereotaxic coordinates interaural 1.5 to 1.7 and bregma −2.1 to 2.3, in regions identified by comparison to a mouse brain atlas. Astrocytes and microglia were visualized in the cornu ammonis (CA) 1, CA2, CA3, and the dentate gyrus (DG), while FJC-positive neurons were counted in the CA1 and DG regions both ipsilateral and contralateral to the injection site. Cells were visualized (0.83 mm × 0.66 mm field of view) using epifluorescent microscopy. Iba1-labelled microglia and GFAP-labelled astrocytes were counted using Nikon Elements software (NIS-Elements Basic Research, version 3.1) as described previously [57] with a ×10 objective. Images were acquired using automated exposure, and the fluorescence intensity for each image was manually adjusted to fall within the linear range. Three operations were applied to the images: ×6 clean, ×4 separate, and ×0 smooth. Counting in Nikon Elements was performed by an experimenter (KEH) self-blinded (by randomly assigning slices numbers 1, 2, 3…*n* prior to each immunohistochemistry run) to the genotype/diet grouping and time point, and all images in the iba1 channel were counted a second time for validation by a second experimenter (VG), blinded by the same method, using ImageJ software by manually thresholding the image and using the Analyze Particles plugin with a size exclusion limit of 40 μm^2^. FJC-positive neurons in the CA1 and DG regions of the hippocampus were counted manually in three predetermined 150 × 150 μm boxes per image, and scores were validated in a subset of samples by a second blinded observer.

### Confocal microscopy and microglia morphology

**Fig. 2:**
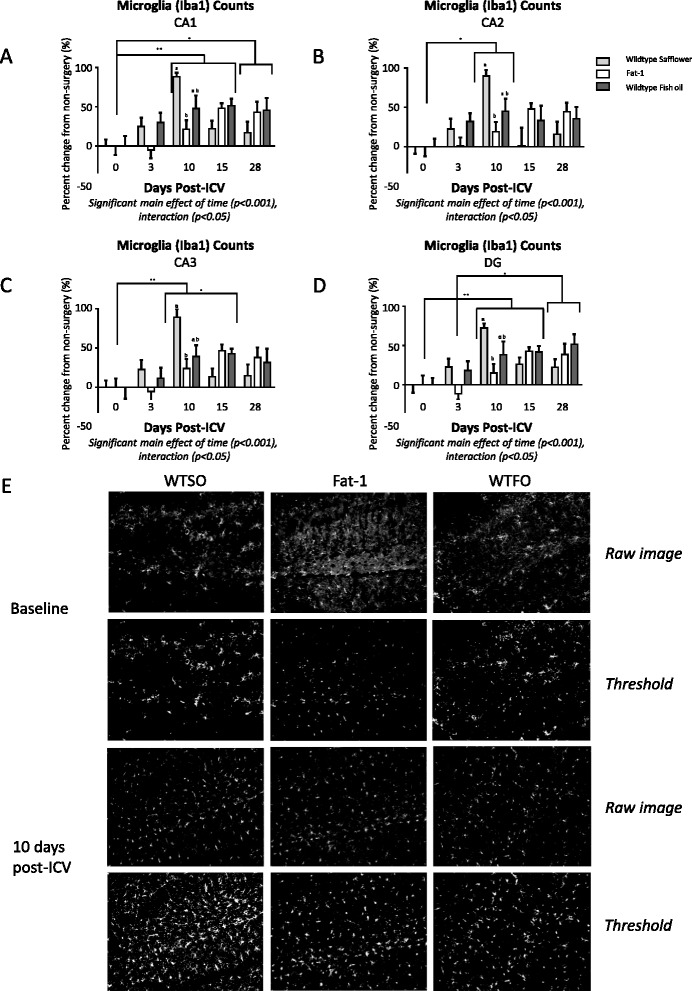
**a**–**d** Iba1-labelled microglia cell counts (±SEM) normalized to non-surgery counts in the hippocampus regions CA1 (**a**), CA2 (**b**), CA3 (**c**), and dentate gyrus (**d**). **e** Representative images of the CA1 region of wild type mice fed safflower oil (WTSO), fat-1 transgenic mice fed safflower oil (fat-1), and wild type mice fed fish oil (WTFO) prior to surgery (baseline, *top two rows*) and at 10 days post intracerebroventricular infusion of amyloid-β peptide (*bottom two rows*). The *top rows* for each time point are images enhanced for contrast and sharpness for publication; *bottom images* are the same images in which a threshold was applied to show labelled cells. Two-way ANOVA was applied; significant main effects and interactions are reported beneath each graph. *Different letters* denote significantly different bars within a time point (e.g., 10 days post-surgery), while *lines* and *asterisks* indicate overall differences between time points. *p < 0.05, **p < 0.01. *CA* cornu ammonis, *DG* dentate gyrus, *iba1* ionized calcium-binding adapter molecule 1. *SEM* standard error of the mean

**Fig. 3:**
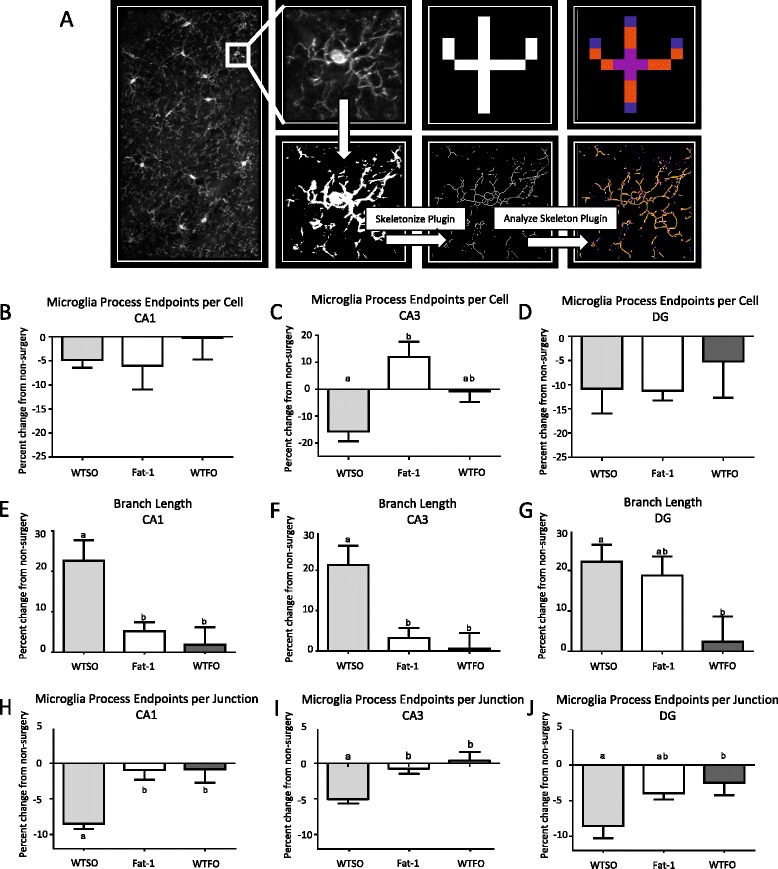
**a** Schematic illustrating the method for measuring microglia morphology with ImageJ Analyze Skeleton (reproduced in part with permission). Confocal z-stacks are converted to maximum intensity projections, and then thresholded to create a binary image. Images were converted into 2D skeletons by the Skeletonize 2D/3D plugin, and pixels were analyzed by the Analyze Skeleton plugin. Pixels with one neighbor (labelled in *blue*) are branch end points, pixels with two neighbors (labelled in *orange*) are branches or slabs, and pixels with three or more neighbors (labelled in *pink*) are junctions. Average number of microglia process end points (an indicator of the number of microglia processes) per cell (**b**–**d**), average process length per cell (**e**–**g**), and process end points per junction, used here as an indicator of branching complexity (**h**–**j**) in the CA1, CA3, and DG. All graphs represent values at 10 days post-intracerebroventricular infusion of amyloid-β 1-40 normalized for non-surgery values, ±SEM. *Different letters* denote significantly different bars (*p* < 0.05) as determined by one-way ANOVA with Bonferroni post-test. *CA* cornu ammonis, *DG* dentate gyrus, *WTSO* wild type mice fed safflower oil, *fat-1* fat-1 mice fed safflower oil, *WTFO* wild type mice fed fish oil. *SEM* standard error of the mean

As microglia are thought to take on an amoeboid morphology, characterized by fewer, less complex branches and a larger cell body upon activation, brain sections at baseline and 10 days post-surgery (a peak point in microglia activation, Fig. 2) were analyzed by confocal microscopy and skeleton analysis to assess microglia morphology. Twenty micrometer z-stacks of CA1, CA3, and DG in both the left and right hippocampus were acquired at 0.5 μm intervals using an AxioObserverZ1 spinning disk confocal microscope (Zeiss, Oberkochen, Germany) at ×20 objective. Microglia morphology was measured in all cells (0.44 mm × 0.25 mm field of view) in each region using a method adapted from Morrison et al. [58]. As illustrated in Fig. 3a, maximum intensity projections for the Iba1 channel of each image were generated, binarized, and skeletonized using the Skeletonize 2D/3D plugin in ImageJ, after which the Analyze Skeleton plugin (http://imagej.net/AnalyzeSkeleton) was applied with the lowest intensity voxel prune cycle. This plugin analyzes the pixels of each skeletonized microglia and categorizes them based on their relationship to one another, with pixels with only one neighboring pixel considered end points, pixels with two neighbors considered slabs (or in this case, a branch), and pixels with more than two neighbors considered junction points. The average branch number (process end points per cell) and length per cell was recorded for each image with a voxel size exclusion limit of 150 applied. The ratio of end points to junction points was additionally calculated to give an indication of branching complexity.

### Statistical analysis

**Fig. 4:**
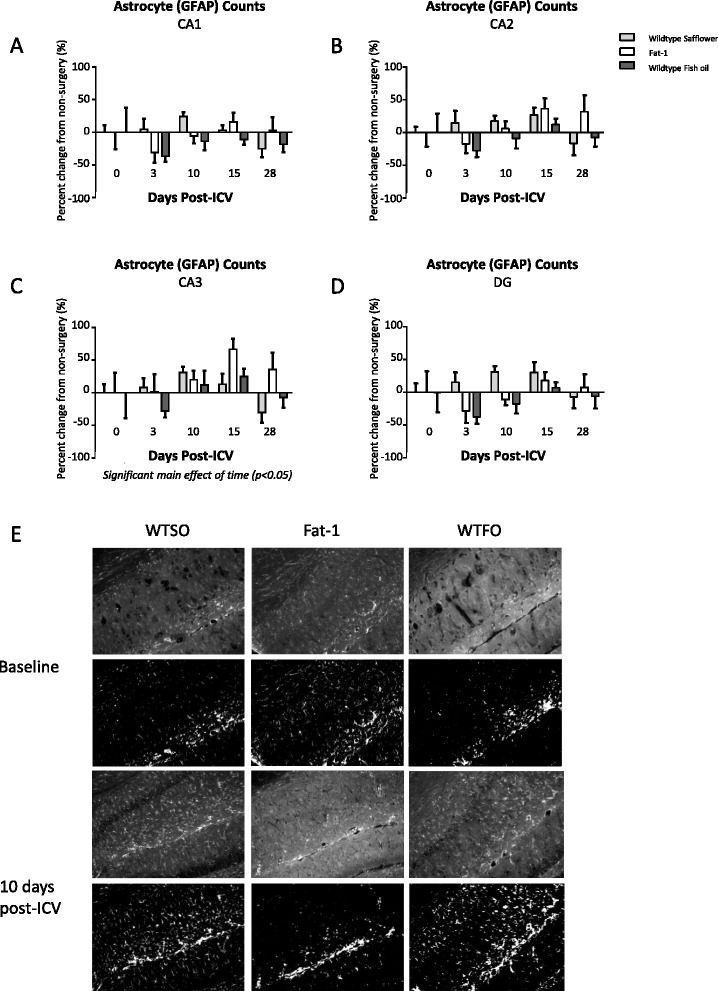
**a**–**d** GFAP-labelled astrocyte cell counts (±SEM) normalized to non-surgery counts in the hippocampus regions CA1 (**a**), CA2 (**b**), CA3 (**c**), and dentate gyrus (DG) (**d**). **e** Representative images of the CA1 region of wild type mice fed safflower oil (WTSO), fat-1 transgenic mice fed safflower (fat-1), and wild type mice fed fish oil (WTFO) prior to surgery (baseline, *top two rows*) and at 10 days post-intracerebroventricular infusion of amyloid-β peptide (*bottom two rows*). The *top rows* for each time point are images enhanced for contrast and sharpness for publication; *bottom images* are the same images in which a threshold was applied to show labelled cells. Two-way ANOVA applied, where significant main effects are reported beneath each graph. *CA* cornu ammonis, *DG* dentate gyrus, *GFAP* glial fibrillary acidic protein. *SEM* standard error of the mean

Data are expressed as mean ± standard error of the mean, normalized to control peptide-injected animals (amyloid-β 40-1) in Fig. 1, or to non-surgery animals of their treatment group in Figs. 2 and 4. Cell counts for each hippocampal region were obtained from one slice per animal in the left, and right sides of 3–5 brains per treatment per time point were used for the work in the C57BL/6 mice, and in 6–12 brain samples per treatment/genotype per time point in the work with fat-1 mice and their wild type littermates. One-way analysis of variance (ANOVA) with a Bonferroni post-test was applied to evaluate differences by genotype/treatment group in brain fatty acid composition, FJC counts, and microglia morphology at 10 days post-icv, while a two-way ANOVA was used to examine main and interactive effects of genotype/treatment groups and time, with a Bonferroni post hoc test applied where there was a significant interaction.

## Results

### Time course of microglia and astrocyte activation following icv amyloid-β 1-40 or control peptide

We first set out to identify time points at which to visualize the neuroinflammatory response to amyloid-β 1-40 relative to a control peptide, amyloid-β 40-1. Aggregation of amyloid-β 1-40 was confirmed by electron microscopy (Fig. 1a) by identifying fibrils 100–500 nm long and smooth in appearance [54, 55]. Increases in iba1-labelled microglia were seen in the days following surgery in all four regions of the hippocampus measured relative to control (Fig. 1b, graph shown for the mean of four fields), and counts were significantly different from 24 h at 15 days (CA2, CA3, and DG) and 21 days (CA1, CA2, and CA3) post-icv. Peak levels of microglia were approximately 50 % greater in the animals injected with amyloid-β 1-40 as opposed to the control peptide injected animals at 15 days post-icv. In all regions, counts were no longer different from 24 h or the peak by 28 days following surgery.

Increases in GFAP-labelled astrocytes (Fig. 1d, graph shown for the mean of four fields) were detected in the CA1, CA2, and CA3 regions, with a peak at 15 days post-surgery that was significantly different from 24 h. In all three regions where this pattern was detected, no significant difference between the peak and its baseline was seen by 28 days post-surgery. No significant differences were detected between any of the time points in the DG.

### Effect of brain fatty acid composition on time course of microglia and astrocyte activation

**Fig. 5:**
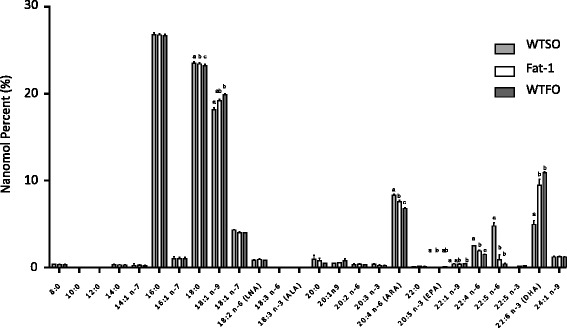
Whole brain fatty acid composition (±SEM) of wild type mice fed safflower oil (WTSO), fat-1 transgenic mice fed safflower oil (fat-1), and wild type mice fed fish oil (WTFO). *Bars* illustrate nanomolar percent of all detected fatty acids in the brain. One-way ANOVA applied for each fatty acid and, where significant, followed by a Bonferroni post hoc test. *Different letters* denote significantly different means, *p* < 0.05. *ALA* alpha-linolenic acid, *LNA* linoleic acid, *ARA* arachidonic acid, *EPA* eicosapentaenoic acid, *DHA* docosahexaenoic acid

The effect of brain omega-3 PUFA composition on the neuroinflammatory response to amyloid-β was assessed in fat-1 mice and their wild type littermates weaned onto either a 10 % safflower oil diet, containing very low levels of omega-3 PUFA, or a diet in which 2 % of the safflower oil was replaced with fish oil. A dietary and a transgenic approach to increasing brain omega-3 PUFA was used to account for potential confounding arising from either off-target effects of the transgene, in the case of the fat-1 mice, or from changes in other elements of the diet in the case of the WTFO mice, as adding 2 % fish oil involves the removal of 2 % safflower oil. Fat-1 and WTFO mice had approximately twofold higher levels (*p* < 0.05) of brain DHA as a nanomolar percent of fatty acids relative to wild type safflower fed (WTSO) mice (Fig. 5). They also had significantly lower levels of the omega-6 PUFA: arachidonic acid, docosapentaenoic acid, and docosatetraenoic acid. As a result, the fat-1 and WTFO mice had a two to threefold lower ratio of brain omega-6: omega-3 PUFA than WTSO mice.

As earlier experiments showed that the injection of amyloid-β 1-40 is more potently neuroinflammatory than the control peptide (Fig. 1), microglia and astrocyte counts were normalized to non-surgery animals for each diet/genotype group rather than to control peptide-injected animals for each time point in an effort to reduce the number of animals required for this study. Microglia activation of the mean of both left and right hippocampus regions CA1, CA2, CA3, and DG peaked at 10 days post-surgery, and for CA2, CA3, and DG, were no longer significantly different from baseline levels by 28 days (Fig. 2a–d). A two-way ANOVA (genotype/diet × time) returned a significant main effect of time (*p* < 0.001) in all four regions with a significant interaction (*p* < 0.05). Post hoc analysis of the treatment effect within each time point showed that fat-1 mice had a lower peak in iba1-labelled microglia number at 10 days post-surgery than the WTSO mice, while WTFO mice were not different from either group at any time in any region. Post hoc analysis of the time effect showed that microglia counts for the three groups were significantly higher than baseline levels at 10 days (CA1, CA2, CA3, and DG) and 15 days (CA1 and DG) post-icv. Counts remained elevated from baseline at 28 days in the CA1 and elevated compared to 3 days in the DG. When the highest count rather than the average of the left and right regions was used for analysis, the significance of the interaction effect was lost in CA1 and CA3.

When the mean of GFAP-labelled astrocyte counts in the left and right hippocampus were analyzed (Fig. 4), no significant main effects of genotype/diet, time, or interactions were found in CA1, CA2, or DG, while a significant main effect of time was identified in CA3 (*p* < 0.05). When the side with the highest counts was used for analysis, a significant main effect of time was identified in the CA1, CA2, and CA3 with no genotype/diet × time interaction. The highest levels of astrocyte counts occurred at 10 and 15 days post-icv, with counts up to 50 % above baseline levels.

### Fluoro Jade C cell counts

**Fig. 6:**
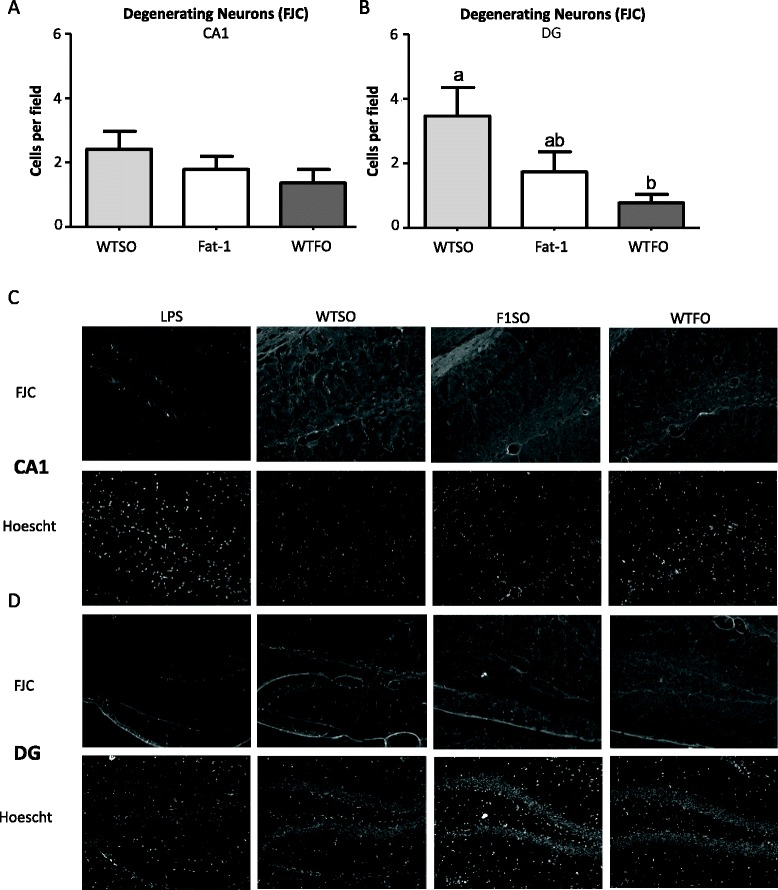
Fluoro Jade C-positive cells (±SEM) in the CA1 (**a**) and DG (**b**) regions of the hippocampus. *Graphs* represent counts (mean of three boxes per image) at 10 days post-icv amyloid-β 1-40. Representative images of the CA1 (**c**) and DG (**d**) of the following: from *left to right*: LPS-injected positive control mice, wild type mice fed safflower oil (WTSO), fat-1 transgenic mice fed safflower (fat-1), and wild type mice fed fish oil (WTFO) mice showing the FJC-positive cells (*top*) and Hoescht staining (a non-discriminate DNA stain). Images are enhanced for contrast and sharpness for publication. All graphs represent values at 10 days post-intracerebroventricular infusion of amyloid-β 1-40 normalized for non-surgery values ±SEM. *Different letters* denote significant differences (*p* < 0.05) as determined by one-way ANOVA with Bonferroni post hoc test. *CA* cornu ammonis, *DG* dentate gyrus, *WTSO* wild type mice fed safflower oil, *fat-1* fat-1 mice fed safflower oil, *WTFO* wild type mice fed fish oil

Degenerating neurons were visualized with FJC immunohistochemistry at 10 days post-icv; the time point at which a difference between the genotype/treatment groups in microglia counts was observed. No difference between the groups was detected in CA1; however, WTFO mice had significantly fewer FJC-positive neurons in the DG compared to the WTSO mice, while fat-1 mice were not different from either group (Fig. 6).

### Microglia morphology

Microglia morphology was investigated to determine whether the differences in cell counts between the diet/genotype groups identified at 10 days post-icv were related to microglia activation. No significant differences between the groups were identified for the number of microglia process end points per cell, used here and previously as an indicator of the number of branches per cell [58, 59], in the CA1 or DG. Microglia of fat-1 mice had on average significantly more end points per cell than the WTSO group, but not the WTFO group in CA3 (Fig. 3b–d). Relative to WTSO mice, microglia process length was lower in fat-1 and WTFO mice in CA1 and CA3, and in WTFO mice alone in DG (Fig. 3e–g). The number of process end points per junction was calculated to give an indication of branching complexity or the number of new branches arising from branch splitting. Both fat-1 and WTFO mice had significantly higher branching complexity following surgery than the WTSO mice in CA1, CA3, and DG (Fig. 3h–j).

## Discussion

Here, we show that the numbers of iba1-labelled microglia and GFAP-labelled astrocytes increase following icv infusion of amyloid-β 1-40, peaking in C57BL/6 mice between 15 and 21 days post-surgery, and in fat-1 and wild type mice at 10 days post-surgery. The inflammatory response resolves in most regions examined by 28 days post-surgery, with cell counts no longer significantly different from baseline levels. There was no effect of the twofold increase in the brain DHA in the fat-1 or WTFO mice compared to WTSO mice on astrocyte counts in the four areas of the hippocampus measured; however, a reduction in the peak in microglia cell number at 10 days post-surgery was noted in fat-1, but not the WTFO mice, compared to the WTSO mice. There was no effect of this change in omega-3 PUFA on the time course of microglia or astrocyte activation up to 28 days following surgery.

There are some indications that microglia in the hippocampi of fat-1 and WTFO mice take on a less activated skeleton structure following icv amyloid-β than the WTSO mice. As microglia become activated (switching from a surveillance and neurotrophic role to a phenotype in which replication, migration, cytokine production, and phagocytosis occur), they are thought to shift from a ramified appearance, with numerous processes and a small cell body, to an amoeboid phenotype, characterized by fewer, shorter processes and a larger cell body [60]. Fat-1 mice had a smaller reduction in process end points per cell in CA3 in response to icv amyloid-β, while both fat-1 and WTFO mice retained more end points per junction, indicating more branch splitting. A reduction in process end points with microglia activation has been measured using this same method previously in both a stroke [58] and an AD [59] model, while omega-3 PUFA deficiency has previously been shown to alter markers of microglia activation (M1 and M2) and process motility [61]. Surprisingly, branch length increased in our model in response to icv amyloid-β, with greater increases in WTSO mice than in the other two groups, whereas branch length decreased with activation in the previous studies using this method [58, 59]. This unexpected finding could be explained by retractions of terminal branches, leading pixels that were initially counted as multiple separate branches connected by junction points to be counted as a single longer branch, which is supported by the reductions in end points per junction seen in Fig. 3h–j. Thus, the decrease in branch length could be indicative of decreased branching complexity, associated with a more inflammatory phenotype, though this should be examined in other studies. It should be noted that while skeleton analysis has benefits of unbiased batch processing and branch quantification, it does not measure the changes in soma size or branch thickness that may also occur with microglia activation or dystrophia.

While this study provides evidence for a potential mechanism that may underlie the protective effects of omega-3 PUFA in AD that have been observed in human observational studies [35, 36], animal models [37], and on cognitive decline in some human clinical trials (in patients with mild, but not moderate or advanced AD [39]), there are limitations to the interpretation of the results. The icv amyloid-β model was selected instead of a transgenic AD model as it allows for the full dynamic response of microglia and astrocytes to amyloid-β, including an increase in activation followed by resolution to baseline levels, to be measured. This would not be possible with the sustained production of amyloid-β that occurs in transgenic models, as the glia would be continually stimulated. This method is, however, limited in comparison with some transgenic models in its applicability to AD in humans because it relies on an acute as opposed to chronic exposure to amyloid-β and does not take into account the hyperphosphorylation of tau proteins.

Another limitation of this work is that increasing brain DHA via either a dietary or a transgenic approach proportionately reduced brain omega-6 PUFA, resulting in an altered omega-6:omega-3 ratio. It is possible, therefore, that some of the immunemodulatory effects reported here could be attributed to the reduction in omega-6 PUFA, rather than the increase in omega-3 PUFA. This proportional change in brain omega-6 PUFA would arise in any intervention aimed at increasing brain omega-3 PUFA, so this confounder does not diminish the clinical or biological relevance of the findings of this paper. To our knowledge, no one has yet investigated the effects of modulating dietary omega-6 PUFA on neuroinflammation in an AD model, though lowering dietary linoleic acid has recently been shown to attenuate the increase in prostaglandin *E*_2_, a pro-inflammatory lipid mediator derived from arachidonic acid, and the activity of cyclooxygenase (COX)-2, an enzyme involved in its synthesis, in response to icv LPS [62].

A difficulty in testing for potential anti-neuroinflammatory effects of omega-3 PUFA is that, in addition to modulating inflammation, these fatty acids are also known to be neuroprotective, decreasing the magnitude of neurological insult associated with a disease model. For example, administration of DHA 3 h after medial cerebral artery occlusion, a model of stroke, decreases infiltration of microglia/macrophage lineage cells, but also decreases the volume of the ischemic infarct [63]. The reduction in microglia infiltration in these models could be explained either by a direct effect of DHA treatment on microglia or as a lower level response to a smaller injury. The same concerns arise in transgenic models of AD, where DHA decreases the amyloidgenic processing of amyloid precursor protein [64], leading to decreased production of plaque-forming amyloid-β both in vitro [65] and in vivo [66]. The icv amyloid-β model used here avoids some of this confounding by administering exogenous amyloid-β; however, confounding is not entirely removed because fewer FJC-positive neurons were detected in one region of the hippocampus of WTFO mice compared to WTSO mice (Fig. 6), indicating less neuronal degeneration. It is possible then that some of the differences in microglia cell number and morphology reported here could be explained as a decreased response to a smaller neurological insult, rather than a direct effect of brain PUFA on the activation of these cells. However, because the fat-1 group had the smallest increase in microglia cell number in response to amyloid-β but the WTFO group had the lowest level of FJC-positive neurons, these results do not appear to be directly correlated.

While increasing brain DHA may attenuate the increase in microglia counts and alterations in microglia morphology in response to icv amyloid-β, it is not known whether these differences are of a sufficient magnitude to be functionally relevant, and if they are, whether they would be beneficial in AD. While several groups have noted improvements in cognition and neuronal death in association with reductions in inflammatory markers, including microglia activation, in AD [67, 68], this is not consistent across studies. For instance, Michaud et al. noted improvements in amyloid-β clearance and cognition in a transgenic model of AD in response to an agonist of the TLR4 receptor, which activates microglia [69]. Chakrabarty et al. separately overexpressed IL-6 [70] and IFN-γ [71] in TgCRND8 mice and measured lower levels of amyloid-β plaque deposition despite elevations in markers of astrocytes and microglia, suggesting that increases in these cells may in fact be protective, at least at an early stage in the disease. In contrast, omega-3 PUFA, which have been shown here and in other studies to be anti-inflammatory in AD models [42, 43], appears to exert protective effects on neuronal loss, amyloid burden, and cognition [37]. Part of the discrepancy between these models may be related to the functional effectiveness of microglia in AD. While an amoeboid phenotype is classically associated with microglial activation, it is now known that amyloid-β contributes to microglial dysfunction, including decreased phagocytic capacity, so that microglia in AD may be phenotypically, but not functionally, activated [60, 72]. Increasing activation of microglia may be beneficial acutely in AD models by increasing clearance of amyloid-β; however, as microglia become dysfunctional due to exposure to amyloid-β, the elevated numbers seen in human AD and animal models may instead contribute to neuronal death and dysfunction. As omega-3 PUFA promote amyloid-β phagocytosis by microglia and increase the expression of phagocytic markers [34, 73], they may prevent this dysfunction in microglia activity, allowing microglia to exert beneficial effects in AD.

In this study, differences in the time course of the astrocyte and microglia responses to amyloid-β were noted between the experiments conducted in C57BL/6 mice and the fat-1 strains. Counts for both astrocytes and microglia were generally lower and peaked later in the C57BL/6 mice, at 15 and 21 days post-icv, respectively, compared to at 10 days in the fat-1 study. In addition, there was a main effect of time on astrocyte number in all four regions of the hippocampus measured in the study using C57BL/6 mice, while in the experiment using the fat-1 mice, a main effect of time was evident in CA3 but not CA1, CA2, or DG. Fat-1 mice have a C57BL/6 and C3H strain background, and previous work in our lab found that fat-1 progeny are 76 % genetically similar to C57Bl/6 mice [49]. Therefore, genetic differences between the mice in the two studies could explain some of these discrepancies. Another possibility is that dietary differences exerted an effect. The C57BL/6 mice were maintained on a chow diet containing 6 % ALA, the precursor to DHA, for the duration of the experiment while the fat-1 study used a safflower oil diet containing <1 % ALA. A diet containing 200 mg/100 g diet of ALA (2–2.5 % of fatty acids on a 10 % fat diet) is thought to be sufficient to maintain brain DHA levels [74], so these two studies differ not only in the DHA content of the diets but also in the sufficiency of the ALA content to maintain brain DHA levels. A further possibility is that experimental differences contributed to the differences in the results between these two studies. These experiments were conducted at different times, and while efforts were made to keep the protocols consistent, different stocks of reagents (including a new batch of amyloid-β) and new versions of analysis software were used. Thus, caution must be undertaken in directly comparing the results of the experiments in the C57BL/6 and fat-1 mice.

Differences were also noted between the fat-1 and WTFO mice in the microglia response to amyloid-β, despite these groups having similar brain levels of DHA. Fat-1 mice had significantly lower numbers of iba1-labelled microglia 10 days post-surgery than the WTSO mice, while the WTFO mice were not significantly different from either group. In contrast, WTFO, but not fat-1, mice had significantly fewer degenerating neurons following icv amyloid-β than WTSO mice. In the previous work, from our lab using fat-1 mice and the same diets, fat-1 mice exhibited enrichment in the brain unesterified DHA compared to wild type mice fed with a safflower oil diet, while C57BL/6 mice fed with the fish oil diet did not demonstrate this comparative enrichment relative to C57BL/6 mice fed with the safflower oil diet [49]. In this study, the neuroinflammatory response to LPS was attenuated in the fat-1 group, but not the fish oil group compared to safflower fed mice, and this was attributed to the differences in unesterified DHA [49]. Unesterified fatty acids are substrates for the synthesis of specialized pro-resolving lipid mediators [29], so it follows that changes in the unesterified pool may be more functionally important than changes in the whole brain. Fat-1 and WTFO mice also differ in the duration of exposure to omega-3 PUFA and DHA. As fat-1 mice produce omega-3 PUFA endogenously, they are exposed to these fatty acids throughout gestation and growth, while WTFO mice are exposed only after weaning. Thus, differences between these groups of mice could also be attributed to early programming of the inflammatory response due to exposure to omega-3 PUFA during critical periods of development. It should be noted, however, that the direction of effect was always the same for the fat-1 and WTFO groups and these groups were never significantly different from one another. This consistency suggests that the inflammation attenuating effects observed in the fat-1 and WTFO mice is attributable to the common change in the brain fatty acid composition that occurred in both groups and not due to residual confounding by diet or genotype. Brain fatty acid uptake, turnover, and metabolism could differ between diet/genotype groups and with surgery, so lipidomics and fatty acid kinetics in response to inflammation should be investigated in future studies to offer mechanisms by which changing brain omega-3 PUFA composition affects neuroinflammation.

## Conclusions

Increasing brain omega-3 PUFA, through transgenic, and to a lesser extent, through dietary means decreased microglia responses to amyloid-β infusion in a mouse model of AD, though no effects on astrocyte number, or the length of time for microglia activation to resolve to baseline levels were evident. Omega-3 PUFA have been shown in many human observational and animal studies to be protective against AD symptoms and pathology, and this study provides evidence that this may occur through modulation of neuroinflammation, though further work is needed to test this hypothesis directly.

## Abbreviations

AD: Alzheimer’s disease
ALA: Alpha-linolenic acid
ANOVA: Analysis of variance
CA: Cornu ammonis
CD: Cluster of differentiation
COX: Cyclooxygenase
DG: Dentate gyrus
DHA: Docosahexaenoic acid
EPA: Eicosapentaenoic acid
FJC: Fluoro Jade C
GFAP: Glial fibrillary acidic protein
iba1: Ionized calcium-binding adapter molecule 1
icv: Intracerebroventricular
IFN: Interferon
IL: Interleukin
MCP: Monocyte chemoattractant protein
PBS: Phosphate-buffered saline
PIB: Pittsburgh compound B
PPAR: Peroxisome proliferator-activated receptor
PUFA: Polyunsaturated fatty acids
SEM: Standard error of the mean
TLR: Toll-like receptor
TNF: Tumor necrosis factor
TREM: Triggering receptor expressed on myeloid cells
WTFO: Wild type fed fish oil
WTSO: Wild type fed safflower
